# Obesity and prostate cancer: gene expression signature of human periprostatic adipose tissue

**DOI:** 10.1186/1741-7015-10-108

**Published:** 2012-09-25

**Authors:** Ricardo Ribeiro, Cátia Monteiro, Victoria Catalán, Pingzhao Hu, Virgínia Cunha, Amaia Rodríguez, Javier Gómez-Ambrosi, Avelino Fraga, Paulo Príncipe, Carlos Lobato, Francisco Lobo, António Morais, Vitor Silva, José Sanches-Magalhães, Jorge Oliveira, Francisco Pina, Carlos Lopes, Rui Medeiros, Gema Frühbeck

**Affiliations:** 1 Molecular Oncology Group, Portuguese Institute of Oncology, Ed. Laboratórios-Piso 4, Rua Dr. António Bernardino de Almeida 4200-072, Porto, Portugal; 2 ICBAS, Abel Salazar Biomedical Sciences Institute, University of Porto, Rua de Jorge Viterbo Ferreira nº 228, 4050-313, Porto, Portugal; 3 Metabolic Research Laboratory, Clínica Universidad de Navarra, Pío XII 36, 31008, Pamplona, Spain; 4 LPCC - Portuguese League Against Cancer (NRNorte), Est. Interior da Circunvalação 6657, 4200-177, Porto, Portugal; 5 CIBER Fisiopatología de la Obesidad y Nutrición, Instituto de Salud Carlos III, Pamplona, Spain; 6 The Center for Applied Genomics, Hospital for Sick Children, MaRS Centre - East Tower 101 College Street, Room 15-705, Toronto, Ontario, M5G 1L7, Canada; 7 Urology Department, Porto Hospital Centre, Largo Prof. Abel Salazar 4099-001, Porto, Portugal; 8 Urology Department, D. Pedro V Military Hospital, Av. da Boavista 4150-113, Porto, Portugal; 9 Urology Department, Portuguese Institute of Oncology, Rua Dr. António Bernardino de Almeida 4200-072, Porto, Portugal; 10 Urology Department, S. João Hospital, Al. Prof. Hernâni Monteiro 4200 - 319, Porto, Portugal; 11 CEBIMED, Faculty of Health Sciences of Fernando Pessoa University, 4200-150, Porto, Portugal; 12 Department of Endocrinology & Nutrition, Clínica Universidad de Navarra, Pío XII 36, 31008, Pamplona, Spain

**Keywords:** adipose tissue, gene expression, microarray, obesity, periprostatic, prostate cancer

## Abstract

**Background:**

Periprostatic (PP) adipose tissue surrounds the prostate, an organ with a high predisposition to become malignant. Frequently, growing prostatic tumor cells extend beyond the prostatic organ towards this fat depot. This study aimed to determine the genome-wide expression of genes in PP adipose tissue in obesity/overweight (OB/OW) and prostate cancer patients.

**Methods:**

Differentially expressed genes in human PP adipose tissue were identified using microarrays. Analyses were conducted according to the donors' body mass index characteristics (OB/OW versus lean) and prostate disease (extra prostatic cancer versus organ confined prostate cancer versus benign prostatic hyperplasia). Selected genes with altered expression were validated by real-time PCR. Ingenuity Pathway Analysis (IPA) was used to investigate gene ontology, canonical pathways and functional networks.

**Results:**

In the PP adipose tissue of OB/OW subjects, we found altered expression of genes encoding molecules involved in adipogenic/anti-lipolytic, proliferative/anti-apoptotic, and mild immunoinflammatory processes (for example, *FADS1*, down-regulated, and *LEP *and *ANGPT1*, both up-regulated). Conversely, in the PP adipose tissue of subjects with prostate cancer, altered genes were related to adipose tissue cellular activity (increased cell proliferation/differentiation, cell cycle activation and anti-apoptosis), whereas a downward impact on immunity and inflammation was also observed, mostly related to the complement (down-regulation of *CFH*). Interestingly, we found that the microRNA *MIRLET7A2 *was overexpressed in the PP adipose tissue of prostate cancer patients.

**Conclusions:**

Obesity and excess adiposity modified the expression of PP adipose tissue genes to ultimately foster fat mass growth. In patients with prostate cancer the expression profile of PP adipose tissue accounted for hypercellularity and reduced immunosurveillance. Both findings may be liable to promote a favorable environment for prostate cancer progression.

## Background

Prostate cancer is the most common solid neoplasm and the second cause of cancer death in men in Europe [1]. Age, ethnic background and family history are well-established risk factors. In addition, accumulating evidence over the last years has shown that obesity is a relevant risk factor for many types of malignancies, including aggressive prostate cancer [2,3].

Adipose tissue dysfunctional behavior, often seen in obesity, has been widely appreciated as a major cause underlying cancer [4]. The prostate has a capsular-like structure and is surrounded by adipose tissue. Frequently, prostate tumor cells infiltrate the periprostatic (PP) fat pad by transposing or infiltrating the capsule [5], resulting in immediate proximity to adipose tissue. Once cancer cells extend beyond the capsule, the PP adipose tissue-secreted factors, extracellular matrix components or direct cell-cell contact may influence the phenotypic behavior of malignant cells. In fact, recent findings in PP adipose tissue showed that tumor-derived factors influence its metabolic activity profile, and that increased local production of adipokines and PP fat thickness were associated with prostate cancer aggressiveness [6-9]. Furthermore, while the PP adipose tissue gene expression profile is currently unknown, it is well established that adipose tissue from distinct anatomical origins and obesity status has specific gene expression signatures [10,11]. Knowledge of the PP adipose tissue genomic profile may uncover molecules and mechanisms linked either with obesity or prostate cancer that can influence prostate cancer progression.

In this study, we aimed to determine the spectrum of genes differentially expressed in PP adipose tissue as well as relevant functional clustering, in order to evaluate the influence of obesity/overweight (OB/OW) on prostate cancer and vice versa.

## Methods

### Patients

Patients scheduled for retropubic radical prostatectomy or partial open prostatectomy between May and October 2009, without major co-morbidities, were included in this study after they gave informed consent. Inclusion criteria were age (45 to 75 years) and absence of previous prostatic treatments. Exclusion criteria were: diabetes, family history of prostate cancer, transvesical partial open prostatectomy, other primary malignancies, or pharmacological treatment with drugs that may modify adipose tissue gene expression (for example, anti-dislipidemics or anti-diabetics).

Anterior-lateral samples of PP adipose tissue were collected during surgery. Adipose tissue samples were immediately sectioned, cleaned and rinsed with pre-warmed PBS and immersed in RNAlater (Applied Biosystems, Foster City, CA, USA).

Eighteen patients participated in the study and were divided into three groups based on post-surgical diagnosis and pathologic analyses. Six patients with benign prostatic hyperplasia (BPH) and twelve with prostate cancer (six with pT1-T2, organ confined prostate cancer (OCPCa) and six with pT3-T4, extra-prostatic prostate cancer (EPCa)) met the criteria for inclusion in this study. In each prostatic disease group, three patients were lean (body mass index, BMI <25 kg/m^2^) and three were obese/overweight (OB/OW, BMI ≥25 kg/m^2^), resulting in overall nine lean and nine OB/OW. Participants' clinicopathological characteristics and serum PSA concentration at diagnosis were reviewed from clinical charts and are presented in Table 1. The project was approved, from an ethical and scientific standpoint, by the Ethical Committees responsible for research at all institutions, namely the Portuguese Institute of Oncology, Porto Hospital Centre and Porto Military Hospital in Portugal, as well as that of the Clínica Universidad de Navarra in Spain. All reported investigations were carried out in accordance with the principles of the Declaration of Helsinki as revised in 2008.

**Table 1:** Characteristics of participants included in the study

	BPH (n = 6)	OCPCa (n = 6)	EPCa (n = 6)	*P *
Age (years)	67.4 ± 3.9	59.4 ± 2.9	66.9 ± 2.3	0.140^a^
BMI (kg/m^2^)	26.5 ± 1.5	25.2 ± 1.3	26.5 ± 1.6	0.783^a^
Serum total PSA (ng/mL)	8.9 ± 4.7	6.7 ± 1.6	14.7 ± 3.0	0.114^b^
Prostate weight (g)	107.0 ± 12.9	40.8 ± 4.2	71.2 ± 9.5	0.001^a, c^
Serum leptin (mg/mL)	3.7 ± 1.0	7.1 ± 2.5	4.9 ± 1.8	0.453^a^
Combined Gleason score				
≤7 (3+4), n (%)	---	5 (83%)	4 (67%)	1.000^d^
≥7 (4+3), n (%)	---	1 (17%)	2 (33%)	

Continuous variables are presented as mean ± SE. ^a^ANOVA for independent measures; ^b^Kruskal-Wallis test; ^c^LSD post-hoc analysis: BPH versus organ confined PCa, *P *<0.0001, BPH versus extra prostatic cancer, *P *= 0.018, extra prostatic cancer versus organ confined PCa, *P *= 0.040; ^d^Fisher exact test. Median (interquartile range) values for serum total PSA: BPH, 4.7 (3.7 to 18.4) ng/mL; organ confined cancer, 5.1 (4.1 to 10.2) ng/mL; extra-prostatic cancer, 14.7 (8.3 to 21.2) ng/mL. ANOVA, analysis of variance; BMI, body mass index; BPH, benign prostatic hyperplasia; EPCa, extra prostatic cancer; OCPCa, organ confined prostate cancer; PCa, prostate cancer; PSA, prostate specific antigen.

### RNA extraction, microarray hybridization and data processing

Total RNA was extracted from PP adipose tissue samples after homogenization with an ULTRA-TURRAX T25 basic (IKA Werke GmbH, Staufen, Germany) in QIAzol reagent (Qiagen, Valencia, CA, USA) and purified through columns (RNeasy Lipid Tissue Mini kit, Qiagen) with DNase I treatment (RNase-free DNase set, Qiagen). Integrity and purity of RNA were assessed by on-chip electrophoresis using Experion (BioRad, Hercules, CA, USA).

From 1 µg total RNA, cDNA and biotin-labeled antisense cRNA were obtained and hybridized to a high-density oligonucleotide human genome array HG-U133 Plus 2.0 Affymetrix GeneChip Arrays (Affymetrix, Santa Clara, CA, USA). Background correction and normalization were done using a robust multi-array average algorithm [12]. Calculation of fold change values was performed using the lean and the non-cancer or OCPCa as reference. We used Linear Models for Microarray Data [13] to identify differentially expressed genes. Briefly, it starts by fitting a linear model for each gene in the data; then an empirical Bayes method is used to moderate the standard errors for estimating the moderated t-statistics for each gene, which shrinks the standard errors towards a common value. This test is similar to a t-test method for each probe except that the residual standard deviations are moderated across genes to ensure a more stable inference for each gene. The moderated standard deviations are a compromise between the individual genewise standard deviations and an overall pooled standard deviation. We used False Discovery Rate [14] to evaluate the statistical significance of all genes.

### Enrichment analysis using Ingenuity Pathway Analysis software

We evaluated the gene function and network enrichments for selected genes (unadjusted *P *<0.01) using Ingenuity Pathway Analysis (IPA) software (Ingenuity Systems, Redwood City, The OCPCa group of patients was not included in this analysis. Human Genome Organisation (HUGO)-approved gene symbols and their corresponding fold change were uploaded into the software. Networks of these genes were algorithmically generated based on their connectivity and assigned a score. The score takes into account the number of focus genes in the network and the size of the network to approximate its relevance to the original list of focus genes. The identified networks are presented as a figure indicating molecular relationships between genes/gene products. Canonical pathway analysis identified the pathways which were more significant to the input data set.

### Real-Time PCR

To validate the microarray data, a number of representative genes and microRNAs were selected to be studied by real-Time PCR. For gene expression analysis, cDNA was generated using the ThermoScript RT-PCR system (Invitrogen, Carlsbad, CA, USA), whereas for microRNA expression we used the Taqman MicroRNA RT kit (Applied Biosystems), according to the manufacturer's instructions.

Transcript levels of the selected genes and microRNAs were quantified by Real-Time PCR (StepOne, Foster City, CA, Applied Biosystems). The cDNA was amplified using the following conditions, both for genes (Taqman Gene Expression Master Mix, Applied Biosystems) and microRNAs (Taqman Universal Master Mix, Applied Biosystems): 95°C for 10 minutes, followed by 45 cycles of 15 seconds at 95°C and 1 minute at 60°C. Results were normalized to the levels of the *18S *rRNA for genes and of *mir-103 *for microRNA, according to previous reports using adipose tissue [15,16]. Assays' ID are available upon request to the authors. Gene and microRNA expression was calculated using the REST 2009 software, where relative expression was expressed as fold over the reference group. The products of the *PCA3 *gene amplification were verified by 1.5% agarose gel electrophoresis, and acquired using the GelDoc XR system (BioRad) and Quantity One software (BioRad).

### Plasma leptin measurement

Plasma samples were obtained before surgery after an overnight fast. The concentrations of circulating leptin were quantified using microsphere-based multiplexing technology, as previously described [17]. The intra- and inter-assay precisions were 4.2% and 21.4%, respectively. The minimum leptin detectable concentration was 27.4 pg/mL.

### Statistical analysis

Data are presented as mean ± standard error of the mean or median (interquartile range). Departure from normality was tested using the Kolmogorov-Smirnov test. Accordingly, one-way analysis of variance (ANOVA), Kruskal-Wallis or Fisher tests were used for comparisons of clinicopathological variables between prostatic disease groups, whereas differences between OB/OW and lean groups were tested by unpaired t-test, Mann-Whitney and chi-square tests. Data analyses were performed using the software SPSS version 17.0 (SPSS Inc., Chicago, USA) and a *P *<0.05 was considered statistically significant.

## Results

### Patient characteristics

Clinicopathological characteristics of participants according to prostatic disease status are presented in Table 1. Age at diagnosis, BMI, serum leptin and prostate specific antigen (PSA) levels, and combined Gleason grade in subjects with cancer were similar between prostatic disease groups (*P *>0.05 for all comparisons). In this study, as expected, BPH subjects presented heavier prostates (*P *<0.05). The OB/OW had higher mean BMI (29.1 ± 1.8 versus 23.1 ± 1.2 kg/m^2^, *P *<0.0001) and serum leptin levels (8.6 ± 1.3 versus 1.9 ± 0.7 mg/mL, *P *= 0.001) than lean subjects. For each group of prostate disease (BPH, OCPCa and EPCa), significantly higher BMI was observed in OB/OW subjects compared with lean individuals (*P *= 0.016, *P *<0.0001 and *P *= 0.013, respectively).

### PCA3 gene expression in PP adipose tissue

**Figure 1:**
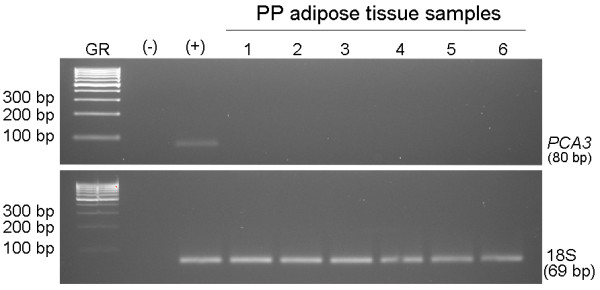
**Reverse transcription PCR analysis of *PCA3 *in PP adipose tissue samples**. GR, gene ruler 100 bp; (+) positive control (prostate tumor sample); (-) negative control (without cDNA); 1-to-6, periprostatic adipose tissue samples of patients with extra prostatic cancer.

Frequently, prostate tumor cells infiltrate the PP fat; therefore, in order to analyze the PP adipose tissue gene expression signature, we needed to confirm the absence of tumor cells. To confirm whether PP adipose tissue samples were free from prostate cancer cells the expression of the *PCA3 *gene was examined. Lack of expression of this gene in the PP adipose tissue of cancer patients was demonstrated (Figure 1).

### Defining the PP adipose tissue gene expression signature in OB/OW subjects

Comparison of overall transcriptional response revealed 148 of the analyzed gene probe sets as significantly changed (*P *<0.001 and absolute fold change ≥1.5). Among these gene probe sets, gene expression features of the overall OB/OW patients associated with six altered genes, whereas OB/OW subjects in restricted BPH, OCPCa and EPCa groups exhibited a specific panoply of altered genes for each condition, which is summarized in Table 2 showing the most representative ones (Table 2).

**Table 2:** Altered genes in microarray analysis in OB/OW PP adipose tissue (overall and by prostatic disease)

Probe set	Gene Name	Gene Description	Fold Change	*P*
All subjects				
8098146	*NPY5R*	neuropeptide Y receptor Y5	1.80	1.49E-05
7948612	*FADS1*	fatty acid desaturase 1	-1.73	1.16E-04
8103494	*NPY1R*	neuropeptide Y receptor Y1	1.97	1.56E-04
7969050	*CYSLTR2*	cysteinyl leukotriene receptor 2	1.89	2.19E-04
8073633	*PNPLA3*	patatin-like phospholipase domain containing 3	-1.56	5.74E-04
7930181	*AS3MT*	arsenic (+3 oxidation state) methyltransferase	1.53	6.54E-04

BPH				
8020343	*ANKRD20A5*	ankyrin repeat domain 20 family; member A5	-1.51	3.95E-05
8152297	*ANGPT1*	angiopoietin 1	1.63	2.08E-04
7937508	*CD151*	CD151 molecule (Raph blood group)	1.69	2.43E-04
7959102	*HSPB8*	heat shock 22kDa protein 8	1.51	3.88E-04
8047780	*SNORA41*	small nucleolar RNA; H/ACA box 41	-1.77	4.94E-04
8098146	*NPY5R*	neuropeptide Y receptor Y5	2.06	7.86E-04
8135909	*LEP*	leptin	1.95	8.00E-04

OCPCa				
**7997239**	***PDXDC2***	**pyridoxal-dependent decarboxylase domain containing 2**	**1.99**	**8.30E-06**
7994026	*NPIPL3*	nuclear pore complex interacting protein-like 3	1.64	1.52E-04
8019280	*PCYT2*	phosphate cytidylyltransferase 2; ethanolamine	-1.54	7.55E-04

EPCa				
7902400	*SNORD45B*	small nucleolar RNA; C/D box 45B	-4.71	2.29E-04
7980861	*CATSPERB*	cation channel; sperm-associated; beta	1.73	5.81E-04
8057004	*PDE11A*	phosphodiesterase 11A	2.59	6.68E-04
8004325	*EIF5A*	eukaryotic translation initiation factor 5A	-1.50	7.72E-04
7969050	*CYSLTR2*	cysteinyl leukotriene receptor 2	2.50	9.01E-04

Fold change and the corresponding *P*-value for each significant gene are presented, using the lean group as reference. The bold ones with adjusted *P *<0.2.

The IPA software was used to investigate functions and interactions among altered genes. This analysis revealed a broad spectrum of biological processes for OB/OW versus lean. Altered functions were predominantly related to nutritional disease, connective tissue development and function, cell death, cellular development and cellular growth and proliferation [See Additional file 1, Table S1]. Additional file 2 Figure S1) shows the most significant network in OB/OW men (*P *<0.0001). In human PP adipose tissue of OB/OW the most relevantly altered canonical pathways were associated with glycerolipid metabolism and leptin signaling (Table 3), and differentially expressed genes encoded proteins involved in immunity and inflammation, cell growth and proliferation, fat metabolism and apoptosis. When restricted to the group of subjects with BPH, being OB/OW was associated with changes in the expression of genes involved in cell-to-cell signaling, tissue development and cellular movement functions [See Additional file 1, Table S1].

**Table 3:** Significant canonical pathways (*P *<0

Pathway	- log_10 _(*P*)
**All (OB/OW versus lean)**	
Glycerolipid metabolism	2.20
Lysine degradation	2.08
Leptin signaling in obesity	1.78

**BPH (OB/OW versus lean)**	
ERK5 signaling	2.31
β-alanine metabolism	1.83
AMPK signaling	1.81
Parkinson's signaling	1.77
C_21_-steroid hormone metabolism	1.77

**EPCa (OB/OW versus lean)**	
Histidine metabolism	1.91
Eicosanoid signaling	1.77
Linoleic acid metabolism	1.73

**All (EPCa versus BPH)**	
Antigen presentation pathway	3.11
Aminosugars metabolism	2.09
B cell development	1.81
OX40 signaling pathway	1.77
ERK5 signaling	1.59
p53 signaling	1.55

**Lean (EPCa versus BPH)**	
T helper cell differentiation	2.24
Galactose metabolism	1.68
Hereditary breast cancer signaling	1.62
Role of osteoblasts, osteoclasts and condrocytes in rheumatoid arthritis	1.51

**OB/OW (EPCa versus BPH)**	
Synaptic long term depression	2.89
Urea cycle and metabolism of aminogroups	2.10
p53 signaling	2.10
Amyothropic lateral sclerosis signaling	2.02
Aminosugars metabolism	1.89
Extrinsic prothrombin activation pathway	1.73
p70S6K signaling	1.68
Cardiac β-adrenegic signaling	1.51

BPH, benign prostatic hyperplasia; EPCa, extra prostatic cancer; OB/OW, obese/overweight.

### Defining the PP adipose tissue gene expression signature in prostate cancer patients

Table 4 shows differentially expressed genes according to prostatic disease (*P *<0.001 and absolute fold change ≥1.5). We found that in the PP adipose tissue of EPCa patients eight genes were up-regulated compared to BPH, while in OCPCa versus BPH most of the genes were down-regulated; a predominance of up-regulated genes was observed in EPCa compared to OCPCa. The PP adipose tissue of cancer patients exhibited increased transcript levels of *MIRLET7A2 *and *TC2N *compared with BPH. Interestingly, *MIRLET7A2 *and *MRPL42 *were overexpressed in all analyses (overall, and within lean and OB/OW groups) in the PP adipose tissue of patients with EPCa. In OB/OW subjects three genes were consistently overexpressed (*TC2N*, *MIRLET7A2 *and *CLDN10*) in the PP adipose tissue of men with cancer (EPCa or OCPCa), compared to BPH.

**Table 4:** Altered genes in microarray according to prostatic disease status (overall, OB/OW or lean group)

Probe set	Gene Name	Gene Description	Fold Change	*P*
**Extra Prostatic Cancer versus Benign Prostatic Hyperplasia**				
All subjects				
**7952313**	***MIRLET7A2***	**microRNA let-7a-2**	**2.00**	**4.26E-06**
**7957540**	***MRPL42***	**mitochondrial ribosomal protein L42**	**1.63**	**5.49E-06**
**7980891**	***TC2N***	**tandem C2 domains; nuclear**	**2.25**	**8.26E-06**
**7915468**	***CCDC23***	**coiled-coil domain containing 23**	**1.70**	**1.93E-05**
8002020	*TPPP3*	tubulin polymerization-promoting protein family member 3	1.66	8.62E-05
7980861	*CATSPERB*	cation channel; sperm-associated; beta	1.84	1.48E-04
7964927	*TSPAN8*	tetraspanin 8	1.91	2.31E-04
8168463	*FGF16*	fibroblast growth factor 16	1.67	2.93E-04

Lean subjects				
7952313	*MIRLET7A2*	microRNA let-7a-2	2.12	9.04E-05
8024485	*GADD45B*	growth arrest and DNA-damage-inducible; beta	-1.56	9.47E-04
7957540	*MRPL42*	mitochondrial ribosomal protein L42	1.60	9.86E-04

OB/OW subjects				
**7980861**	***CATSPERB***	**cation channel; sperm-associated; beta**	**2.50**	**1.91E-06**
**7980891**	***TC2N***	**tandem C2 domains; nuclear**	**2.98**	**4.31E-06**
**7908488**	***CFHR1***	**complement factor H-related 1**	**1.88**	**3.16E-05**
**8078529**	***STAC***	**SH3 and cysteine rich domain**	**1.50**	**5.79E-05**
7902400	*SNORD45B*	small nucleolar RNA; C/D box 45B	-5.18	1.9E-04
7908459	*CFH*	complement factor H	1.70	3.0E-04
7952313	*MIRLET7A2*	microRNA let-7a-2	1.88	4.0E-04
7957540	*MRPL42*	mitochondrial ribosomal protein L42	1.65	4.0E-04
7958019	*DRAM1*	DNA-damage regulated autophagy modulator 1	1.55	5.3E-04
7969640	*CLDN10*	claudin 10	1.59	6.3E-04
8076586	*SCUBE1*	signal peptide; CUB domain; EGF-like 1	-1.73	7.2E-04
8027862	*FFAR2*	free fatty acid receptor 2	-1.58	9.2E-04

**Organ Confined Prostate Cancer versus Benign Prostatic Hyperplasia**				
All subjects				
**7919349**	***RNU1-1***	**RNA; U1 small nuclear 1**	**-2.18**	**1.50E-07**
**7919576**	***RNU1-1***	**RNA; U1 small nuclear 1**	**-1.96**	**2.47E-07**
7952313	*MIRLET7A2*	microRNA let-7a-2	1.79	6.21E-05
7980891	*TC2N*	tandem C2 domains; nuclear	1.98	6.26E-05
7978568	*RNU1-1*	RNA; U1 small nuclear 1	-1.65	9.43E-05
7981964	*SNORD116-8*	small nucleolar RNA; C/D box 116-8	-1.91	1.07E-04
8004184	*XAF1*	XIAP associated factor 1	-1.64	8.67E-04

Lean subjects				
**8001067**	***HERC2P4***	**hect domain and RLD 2 pseudogene 4**	**-1.77**	**1.22E-06**
**7997239**	***PDXDC2***	**pyridoxal-dependent decarboxylase domain containing 2**	**-2.12**	**2.69E-06**
**8000692**	***BOLA2***	**bolA homolog 2 (E, coli)**	**-1.52**	**3.41E-06**
**8000651**	***SMG1***	**SMG1 homolog; phosphatidylinositol 3-kinase-related kinase (C. elegans)**	**-1.61**	**3.76E-06**
**8019655**	***TBC1D3B***	**TBC1 domain family; member 3B**	**-1.56**	**1.26E-05**
**7919349**	***RNU1-1***	**RNA; U1 small nuclear 1**	**-2.30**	**2.27E-05**
**7927513**	***FAM21C***	**family with sequence similarity 21; member C**	**-1.66**	**2.31E-05**
**7993359**	***NPIP***	**nuclear pore complex interacting protein**	**-1.53**	**4.30E-05**
**8014633**	***TBC1D3***	**TBC1 domain family; member 3**	**-1.55**	**5.53E-05**
**8014437**	***TBC1D3G***	**TBC1 domain family; member 3G**	**-1.51**	**6.27E-05**
**7994026**	***NPIPL3***	**nuclear pore complex interacting protein-like 3**	**-1.69**	**9.02E-05**

OB/OW subjects				
**7969640**	***CLDN10***	**claudin 10**	**2.01**	**7.04E-06**
7980891	*TC2N*	tandem C2 domains; nuclear	2.31	1.10E-04
7952313	*MIRLET7A2*	microRNA let-7a-2	2.06	1.74E-04
8021101	*HAUS1*	HAUS augmin-like complex; subunit 1	1.61	3.10E-04
8165698	*MIR1977*	microRNA 1977	-3.03	3.46E-04
8060854	*PLCB1*	phospholipase C; beta 1 (phosphoinositide-specific)	1.53	5.95E-04

**Extra Prostatic Cancer versus Organ Confined Prostate Cancer**				
All subjects				
7919349	*RNU1-1*	RNA; U1 small nuclear 1	1.71	1.86E-05
7981964	*SNORD116-8*	small nucleolar RNA; C/D box 116-8	1.88	1.85E-04
7940287	*MS4A1*	membrane-spanning 4-domains; subfamily A; member 1	-2.15	2.74E-04

Lean subjects				
**8001067**	***HERC2P4***	**hect domain and RLD 2 pseudogene 4**	**2.06**	**5.14E-08**
**8000692**	***BOLA2***	**bolA homolog 2 (E, coli)**	**1.52**	**3.37E-06**
**7997239**	***PDXDC2***	**pyridoxal-dependent decarboxylase domain containing 2**	**2.00**	**6.36E-06**
**8000651**	***SMG1***	**SMG1 homolog; phosphatidylinositol 3-kinase-related kinase (C. elegans)**	**1.57**	**6.75E-06**
**7927513**	***FAM21C***	**family with sequence similarity 21; member C**	**1.74**	**8.44E-06**
**7993359**	***NPIP***	**nuclear pore complex interacting protein**	**1.51**	**5.38E-05**
**7994026**	***NPIPL3***	**nuclear pore complex interacting protein-like 3**	**1.73**	**5.61E-05**
**8119595**	***RPL7L1***	**ribosomal protein L7-like 1**	**1.63**	**5.71E-05**
**7990943**	***GOLGA6L10***	**golgin A6 family-like 10**	**1.55**	**6.08E-05**
7919349	*RNU1-1*	RNA; U1 small nuclear 1	1.93	2.25E-04

OB/OW subjects				
7980861	*CATSPERB*	cation channel; sperm-associated; beta	1.78	2.92E-04
7982006	*SNORD116-29*	small nucleolar RNA; C/D box 116-29	1.77	5.84E-04

The bold ones with adjusted *P *<0.2

In the analysis of prostate cancer versus BPH, OB/OW subjects presented more altered genes in PP adipose tissue. The IPA analysis identified interaction networks between EPCa versus BPH and found that functions were more frequently related to cellular growth and proliferation, cell cycle, apoptosis and cell death, cellular movement and to inflammation and immunity [See Additional file 3, Table S2] (*P *<0.0001). The altered canonical pathways (*P *<0.05) in the PP adipose tissue of EPCa are shown in Table 3.

### Validation of selected genes by real-time PCR

**Figure 2:**
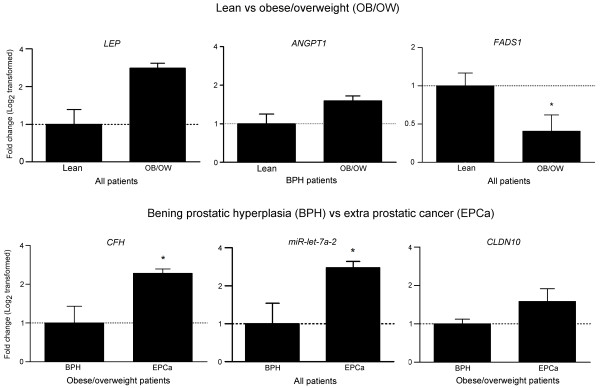
**Validation of selected genes by real-time PCR**. BPH, benign prostatic hyperplasia; EPCa, extra prostatic cancer (≥pT3); Lean (BMI <25 kg/m^2^); OB/OW, obese/overweight (BMI ≥25 kg/m^2^). *ANGPT1*, angiopoietin 1; *CFH*, complement factor H; *CLDN10*, claudin 10; *FAS*, fatty acid desaturase; *LEP*, leptin; *MIRLET7A2*, microRNA let-7a-2. The gene expression in lean subjects or BHP was assumed to be 1. *P <0.05 versus lean or BPH groups.

Validation experiments were performed to confirm the accuracy of array gene expression measurements. We selected a set of representative transcripts involved in cell proliferation, immunity and lipid metabolism. Results across the 18 study patients are shown in Figure 2. Overall, PCR results reflected the findings of microarrays.

## Discussion

Extra-capsular extension of prostate cancer cells into the PP adipose tissue is a common pathologic finding and a factor related with worst prognosis [5]. Once tumor cells extend beyond the prostatic capsule, the interactions with non-tumor cells in the PP adipose tissue may influence its phenotypic behavior. In fact, accumulating evidence shows that the microenvironment is decisive in determining whether cancer cells progress towards metastasis or whether they remain dormant [18]. To date, the scarce reports on PP adipose tissue support a mechanistic link with prostate cancer aggressiveness [6-9].

In the present study, 46% of the well-characterized genes included in the array were expressed at detection level in PP adipose tissue, which is comparable to omental adipose tissue [11]. The comparison of PP adipose tissue gene expression of non-diabetic OB/OW men to lean men with prostatic disease identified, for the first time, 34 differentially expressed genes of which we focused our attention on 20 as the most relevant ones.

Two important but opposed pathways, lipolysis and adipo/lipogenesis, have a significant role in energy balance. In our study, consistent with other reports on visceral adipose tissue [11], altered expression of genes involved in lipolysis were found in association with obesity and excess adiposity. *NPY1R *and *NPY5R *have anti-lipolytic effects [19], whereas *LEP *expression relates to adipo/lipogenesis despite leptin being also a lipolytic factor [20], with all of these overexpressed genes being in the PP adipose tissue of OB/OW men. Noteworthy, *PDE11*, which has been shown to be involved in adipocyte differentiation and cyclic nucleotides biology [21], was overexpressed in PP adipose tissue of OB/OW subjects. Downstream effects of altered genes reportedly up-regulate adipo/lipogenesis, including *PNPLA3 *(also known as adiponutrin), which encodes a triacylglycerol lipase that mediates triacylglycerol hydrolysis [22], *FADS1*, that regulates unsaturation of fatty acids and SREBP-1 expression [23] or *PCYT2*, that mediates phosphatidylethanolamine synthesis and the availability of di- and triacylglycerol [24]. The combined functional dysregulation of these genes suggests that PP adipose tissue from obese men exhibits an anti-lipolytic and adipo/lipogenic gene expression profile.

The number and volume of adipocytes are determinants of fat mass, while proliferation/differentiation and apoptosis influence adipose tissue growth and regression. In our study, the anti-apoptotic genes *ANGPT1 *and *HSPB8 *were up-regulated in PP adipose tissue of OB/OW subjects [25,26]. Furthermore, the expression of *EIF5A*, known to activate the intrinsic mitochondrial pathway [27], was repressed in OB/OW men. Besides altered genes in apoptosis pathways, we found an increased expression of genes involved in cell growth and differentiation, such as *LEP *and *ANGPT1*, whose products increase endothelial, mesenchymal and tumor cell growth and differentiation [25,28-30], and *NPY1R *that mediates a proliferative stimulus in progenitor adipose cells [31]. The data presented here suggest an increased cell growth and anti-apoptosis, extensive to endothelial, progenitor or adult lineages in the PP adipose tissue of OB/OW men.

**Figure 3:**
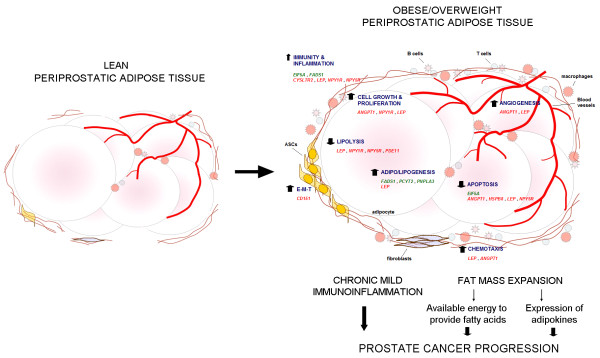
**Schematic summary diagram of PP adipose tissue changes in obese/overweight**. Genes with altered expression are associated with the regulation of functional biological processes. Altered function likely results in chronic mild immunoinflammatory response and fat mass expansion, which ultimately impacts on prostate cancer progression. Down-regulated genes are depicted in green while up-regulated genes are shown in red. ASCs, adipose-derived stem cells; E-M-T, epithelial-to-mesenchymal transition.

Taken together, these anti-lipolytic, adipo/lipogenic, proliferative and anti-apoptotic effects in the PP adipose tissue of OB/OW men likely result in fat mass expansion, conferring increased capacity for enlarged adipocytes to express adipokines and increase fatty acid supplies [32,33], which might impact the local energy and availability of growth factors, thereby causing the local environment to allow cancer progression. This environment in the PP adipose tissue of OB/OW men may, at least partially, explain the described association of obesity and excess adiposity with the progression of prostate cancer [3] (Figure 3). In addition to a local paracrine effect of adipose tissue-derived factors, obesity-related systemic factors may also influence the development of an aggressive phenotype [34].

Recent developments in obesity and cancer immunological pathways suggest a previously unappreciated complexity of cancer cell-adipose tissue cell-immunoinflammatory cell cross-talk [35]. We found altered genes in PP adipose tissue of OB/OW men that are involved in immunity and inflammation. Overexpressed genes engaged in innate and adaptive components of the immune system, include *LEP*, that up-regulates both innate and adaptive immunoinflammatory response [36], *NPY1R*, that has been shown to inhibit T cell activation [37], and *CYSTLR2*, that increases pro-inflammatory cytokine expression [38]. *FADS1*, known to mediate the formation of inflammatory mediators (for example, prostaglandin E2, PGE2, thromboxane A2, TXA2, and leukotriene B4, LTB4) [39], and *EIF5A *that is essential for *NOS2 *translation [40], are both down-regulated in the PP adipose tissue of OB/OW men. Thus, the enhanced local mild immunoinflammatory environment, observed in PP adipose tissue of OB/OW men might further influence tissue remodeling and contribute towards tumor progression.

The *LEP *and *ANGPT1 *encoded proteins may have roles beyond adipose tissue itself. Prostate cancers express the leptin receptor [41] and leptin staining is significantly increased in malignant prostates and poorly differentiated tumors [41]. Also angiopoietin 1 and its receptor Tie-2 were found in both prostate tumor cells and capillaries [42], where they can induce sprouting angiogenesis [43]. These findings along with our own suggest that PP adipose tissue may modulate prostate cancer progression via production of growth factors that favor proliferative and angiogenic events that in turn are needed for tumor development.

Herein, we showed that the PP adipose tissue gene expression profile of OB/OW subjects may contribute to increased local adiposity, a mild immunoinflammatory environment and production of molecules with oncogenic potential (Figure 3).

**Figure 4:**
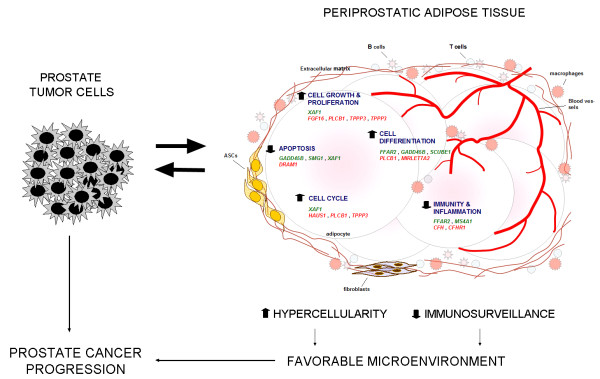
**Schematic summary diagram of changes in PP adipose tissue of patients with prostate cancer**. Periprostatic adipose tissue of patients bearing prostate cancer likely reveals the interactions between tumor cells and adipose tissue cells. The functional repercussions of altered genes in patients with prostate cancer prompt PP adipose tissue to increased hypercellularity and decreased immunosurveillance. The resulting influence of this favorable microenvironment is to foster prostate cancer progression. Down-regulated genes are depicted in green while up-regulated genes are shown in red. ASCs, adipose-derived stem cells.

The PP adipose tissue gene expression signature of men with prostate cancer was determined, in order to determine if functional alterations are associated with prostate cancer besides the previously reported PP thickness and protein measurements [9-12]. In the present study, when the PP adipose tissues of age- and BMI-matched nondiabetic men with different prostatic diseases were compared, 119 altered genes were found. Representative genes and functions are shown in Figure 4. Noteworthy, our findings reveal that altered gene networks pertain to cell cycle and proliferation regulation. Overexpressed genes in the PP adipose tissue of cancer patients that are involved in cell cycle and proliferation include *PLCB1*, that modulates cyclin D3 and CDK4 in response to the IGF-1 mitogenic stimulus [44] or *TPPP3*, that regulates G2-M and G1-S transitions [45]. Furthermore, *HAUS1*, a component of the augmin complex involved in spindle microtubule generation in mitosis [46] and *TSPAN8 *(also known as CO-029), that encodes for an integrin-binding glycoprotein that stimulates endothelial cell proliferation [47], are also up-regulated in cancer patients. Noteworthy, the *FGF16 *gene that encodes a mitogenic growth factor [48] was overexpressed in the PP adipose tissue of men with prostate cancer. These findings, together with the down-regulation of *XAF1*, which influences G2/M arrest through modulation of checkpoint kinase 1 and Cdc2-cyclin B complex [49], support a positive cell cycle regulation and a permissive stimulus for growth and proliferation in PP adipose tissue cells. Cumulatively, pro-apoptotic genes, such as *XAF1 *and *GADD45B *[50,51] were down-regulated, whereas *DRAM1 *was up-regulated in the adipose tissue of prostate cancer patients [52]. Canonical analysis showed involvement of the p53 pathway in adipose tissue of cancer subjects, possibly reflecting the relationship of the altered genes *XAF1*, *DRAM1 *and *SMG1 *with the p53 pathway. In adipose tissue biology, cell differentiation also plays an important role in increasing fat mass. Here we show altered expression of genes that associate with cellular differentiation of overall (for example, *PLCB1*, *GADD45B*), adipocyte (for example, *PLCB1*, *FFAR2*) and endothelial lineages (for example, *SCUBE1*) [44]. Thus, particularly the adipocyte and vascular biology of PP adipose tissue seems to be committed towards the differentiated state in men with prostate cancer. Consistent with gene expression findings, we observed overexpression of the microRNA *MIRLETA2 *in the PP adipose tissue of prostate cancer patients. The involvement of the *let-7 *microRNA in adipocyte differentiation has been described earlier [53]. Moreover, canonical pathways analysis showed that ERK5 signaling, an evolutionary conserved pathway involved in hypertrophic signaling that regulates adipogenic differentiation [54], was altered in PP adipose tissue of cancer patients. Previously, *in vitro *studies showed that tumor-derived factors induce preadipocyte differentiation [55], supporting a tumor-educated regulation of the adipose tissue differentiation program. Taken together, the impact of these gene expression results on cell cycle and proliferation, in apoptosis and differentiation of PP adipose tissue cellular components, supports fat mass accrual, which agrees with findings showing increased PP fat pad thickness in prostate cancer patients [6]. Furthermore, bulky adipocytes predispose to increased adipokine secretion and availability of fatty acids, which may influence prostate cancer progression [32,33].

Immunoinflammatory mechanisms drive both obesity and cancer. Canonical analysis showed that the PP adipose tissue of prostate cancer patients presents altered pathways associated with immunity and inflammation, including antigen presentation, B cell development and T helper cell differentiation. The complement system is important in immunosurveillance against tumors, albeit malignant cells are usually resistant to complement-mediated lysis [56]. The products of *CFH *and *CFHR1 *are soluble complement regulators essential for preventing complement activation and, therefore, responsible for complement inhibition [56]. Interestingly, we and others report on the expression of *CFH *and *CFHR1 *in adipose tissue [57]. The finding of *CFH *and *CFHR1 *up-regulation in PP adipose tissue of subjects with prostate cancer suggests increased inhibitory modulation of the complement activity in prostate tumor cells and evasion to attack. Other altered genes in the PP adipose tissue of prostate cancer patients with repercussion in the local immunoinflammatory environment include *MS4A1 *(also known as *CD20*) that plays a functional role in B-cell activation [58] and *FFAR2 *that encodes a protein reported to modulate the differentiation and/or activation of leukocytes [59]. Taken together, these altered genes in PP adipose tissue might contribute to an environment with immunological cellular dormancy and reduced immunosurveillance, which may facilitate prostate cancer progression (Figure 4).

In the present study, we found increased *CLDN10 *mRNA transcript abundance in the PP adipose tissue of prostate cancer patients using both microarray and real-time PCR analyses. To the best of our knowledge *CLDN10 *expression in adipose tissue has not been previously reported. This gene encodes an important tight junction component with an intriguing role in adipose tissue considering its functions in the stroma arrangement and cellular connections [60]. Further studies are required to obtain insight regarding the cells involved and the functional implications of *CLDN10 *expression in PP adipose tissue.

A global gene expression profiling in PP adipose tissue has been applied for the first time in the present study to unravel genes and regulatory pathways associated with OB/OW and with prostate cancer. Subjects included in this study had histopathological confirmation of prostatic disease, pathology tumor grade and stage, while contamination with prostate tumor cells in the collected PP adipose tissue samples was excluded by the absence of *PCA3 *expression. The high-quality GeneChip data set from RNA specimens of PP adipose tissue, careful patient selection for matching by age, race, BMI and clinical variables underscore the strength of the major findings of this study. However, further research is warranted to uncover the PP adipose tissue gene expression profile in association with distinct obesity grades.

Our findings likely represent the effects of excess adiposity or cancer and the bi-directional interactions between all cell types that influence adipose tissue function and might affect or be influenced by prostate cancer progression. These hypotheses are grounded on the crosstalk between PP adipose tissue and tumor cells, which ultimately may induce an environment favorable to cancer progression. A better understanding of the mechanisms underlying the association between obesity and aggressive prostate cancer is warranted to gain more insight into the specific contribution of each PP adipose tissue cell type to cancer development in order to foster the development of new treatment options and, at the same time, to help identify malignancies with the worst prognosis and encourage the implementation of adjuvant chemoprevention strategies.

## Conclusions

The present study, the first in human PP adipose tissue in which OB/OW and prostate cancer-associated gene expression changes are analyzed by microarrays, provides valuable new insight on how local adipose tissue pathophysiology may favor prostate cancer. We identified altered gene expression that might impact on elements of white adipose tissue overgrowth, including anti-lipolytic, anti-apoptotic, proliferative, and mild local immunoinflammatory stimuli in PP fat of OB/OW subjects. The overexpression of *LEP *and *ANGPT1 *by PP adipose tissue in OB/OW men may contribute towards a favorable environment for prostate cancer progression.

The gene expression signature of PP adipose tissue from prostate cancer patients seems to provide evidence of altered gene expression across distinct cell types, with repercussions on stimuli for cell cycle regulation, cell proliferation and differentiation, as well as anti-apoptosis. Additionally, we found altered genes involved in immunological cell dormancy and reduced immunosurveillance, namely complement-related *CFH *and *CFHR1 *genes. Our findings suggest that the PP adipose tissue gene expression profile of both OB/OW and prostate cancer subjects is likely to cause a local environment favorable to prostate cancer progression. Confirmation of the role of PP adipose tissue in prostate cancer progression together with untangling its mechanisms will become increasingly important in the development of adjuvant therapeutic and lifestyle measures.

## Abbreviations

BMI: body mass index; BPH: benign prostatic hyperplasia; EPCa: extra-prostatic cancer; IPA: Ingenuity Pathway Analysis; OB/OW: obese/overweight; OCPCa: organ confined prostate cancer; PBS: phosphate-buffered saline; PCR: polymerase chain reaction; PP: periprostatic.

## Competing interests

The authors declare that they have no competing interests.

## Authors' contributions

RR, CM, VCa, VCu and AR performed most of the experiments. PH performed the microarray statistical analysis and edited the manuscript. AF, PP, CL, FL, AM, VS, JS-M, JO and FP collected adipose tissue and clinicopathological patient information and edited the manuscript. RR and RM performed the statistical analysis. RR, VCa, JG-A, RM and GF designed the experiments and edited the manuscript. RR and GF obtained the funding for the project. All authors read and approved the final manuscript.

## Pre-publication history

The pre-publication history for this paper can be accessed here:

http://www.biomedcentral.com/1741-7015/10/108/prepub

## Supplementary Material

Additional file 1 **Additional File 1, Table S1**. Significant functions with altered networks and molecules in PP adipose tissue of OB/OW subjects. Click here for file

Additional file 2 **Additional File 2, Figure S1**. Representative network and genes differently expressed in OB/OW versus lean in IPA analysis. Genes are represented as nodes and the biological relationship between two nodes is represented as an edge line. Uncolored genes were not identified as differently expressed in our experiment even though they are relevant to this network. Node shape indicates enzymes (rhombus), phosphatases (triangle), kinases (inverted triangle), G-protein coupled receptor (rectangle), growth factor (square), transporter (trapezoid), transcription factor (ellipse), other (circle). Click here for file

Additional file 3 **Additional File 3, Table S2**. Significant functions with altered networks and molecules in PP adipose tissue of EPCa versus BPH subjects. Click here for file
