# Supplementary material for: Obesity and prostate cancer: gene expression signature of human periprostatic adipose tissue
Source: BMC Med. 2012 Sep 25;10:108. doi: 10.1186/1741-7015-10-108 (PMC3523039; doi:10.1186/1741-7015-10-108)
Supplement: Additional file 1 — , Table S1. Significant functions with altered networks and molecules in PP adipose tissue of OB/OW subjects. [file 1741-7015-10-108-S1.PDF]

Table S1. Significant functions with altered networks and molecules in PP adipose tissue of OB/OW subjects (P<0.0001 for inclusion)

Score	Molecules in Network	Top functions
All subjects		
35	**ACTN1**, **ANGPT1**, Ap1, **APOB**, **CAP2**, **CCND2**, ERK, ERK1/2, FSH, **GDF15**, **GJA1**, GRLF1, hCG, **HSD11B1**, IgG, **ITGB5**, Jnk, **KAT2B**, LDL, **LDLR**, **LEP**, Lh, **LIF**, **MAD2L2**, **MAPK1**, **NPR3**, PDGF BB, **PPAP2A**, **PTPN21**, RNA polymerase II, **SNCG**, **SPARC**, **STS**, **TLK1**, **TRIB1**	Nutritional Disease, Cardiovascular Disease, Connective Tissue Development and Function
24	BUB1B, **DAAM1**, **EIF5A**, **EPB41L4B**, ESR1, FAM125B, FANCC, FGF2, **FHL1**, GLI1, **GRB14**, KDM5B, **LIMCH1**, **MAML2**, **NEDD4L**, NHP2, NOTCH1, **PDLIM4**, **PDZRN3**, **PPP1R12B**, PRDM5, RPL22, RUNX1, SCNN1A, **SETD8**, **SKAP2**, **SNRPE**, **TCEANC**, **TERC**, TERT, TGFB1, **TOM1L1**, TP53, TSG101, **UGT2B7**	Cell Death, Cellular Development, Cellular Growth and Proliferation
17	AGTR1, BCL2L11, CCNB1, CDK1, CDKN1A, **CKAP2**, **CTSB**, **ENDOG**, FOXO3, **GHRL**, Hsp70, ING1, IRF1, **LGALS12**, **MAP1B**, **MAPK1**, **mir-25**, NFIC, **NME5**, NR3C1, **PBRM1**, **PLEKHF1**, **PRKACB**, PRL, PRLR, RB1, RNA polymerase II, ROCK1, SMARCA4, SMARCB1, SNCA, **SOX4**, TP73, **WDR26**, YY1	Cell Death, Cell Cycle, Cellular Development
14	**AGPAT9**, **AMOT**, APOE, BCL6, CCND1, **CCND2**, CDK1, CDKN2A, **DPT**, **EGFL6**, **EIF4EBP1**, **EPB41L2**, **FADS1**, **FASN**, HDAC1, HDAC2, HIC1, **HIPK1**, Histone h4, Hsp70, **LIF**, MBD3 (includes EG:17192), MLH1, NCOR2, NFKB1, PMS2, PP2A, PTTG1, RUNX1T1, SPI1, **THRB**, **TNS3**, TP53, TP63, YY1	Cell Cycle, Cell Death, Tissue Development
14	**AICDA**, **ATAD2**, COL18A1, **COL8A1**, **COPB1**, **ERAP1**, HGF, ID2, IFI16, IRAK1, **IRAK2**, MAVS, MAX, Mek, MYC, MYD88, NFKB1, NFkB (complex), NOS2, **NTRK2**, **PERP**, PIM1, RAC1, **RASA3**, **SETD7**, **SH3RF2**, TAF6, TAF7, TAF10, TBP, TCF3, **TMEM173**, TNFRSF1A, YY1, ZNF217	Gene Expression, Cell Death, Cellular Development
12	ADCY, **ARHGEF6**, ARHGEF7, CCR6, **CLINT1**, CXCR4, **CYSLTR2**, EIF4A1, **EIF4G3**, ELAVL1,	Cell Death, Cell-To-Cell Signaling and

	FOXP3, GIT1, Gm-csf, GNB1, **GNG2**, **HLA-DQB1 (includes others)**, IFNG, IL4, IL23, IL27, IL17A, IL1B, Immunoglobulin, IRS2, **LEP**, **NPY1R**, **NPY5R**, PABPC1, PAK1, PYY, RAF1, RORC, **YAP1**, YWHAB, YWHAG	Interaction, Hematological System Development and Function
Benign Prostatic Hyperplasia		
18	**ADRA1D**, AKAP13, **ANKRD32**, **ARHGEF6**, ARHGEF7, **CD151**, CKAP2, **CLINT1**, **CYP2C9**, **FADS3**, FOXA3, GIT1, HNF4A, ITGA3, ITGB1, **MAP1B**, NFIC, NR1I3, NR3C1, PAX3, **PERP**, **PHLDB2**, PI4KA, ROCK1, RORA, RPS6KA6, SMARCA4, SP1, TCF7, TEAD1, TGS1, TP53BP2, **YAP1**, **YWHAG**, ZAK	Cell-To-Cell Signaling and Interaction, Tissue Development, Cellular Movement
18	**APOB**, CD163, CEBPB, CXCR2, DAB2IP, DEFB4A/DEFB4B, EED, EPC1, EZH2, FANCC, FOXL2, **GRIA1**, HDAC1, HDAC8, HMOX1, HP, Hsp70, **HTRA1**, IL6, IL13, IL22, **MORF4L1**, MRFAP1, NFkB (complex), NFKBIZ, NR5A2, **PCLO**, RB1, **SERPINB2**, **SMG5**, SNX7, SSX2 (includes others), STUB1, **TNS1**, **TOM1L1**	Inflammatory Disease, Cancer, Connective Tissue Disorders
16	**ADM**, **AMOT**, **ANXA2**, APOE, **ATAD2**, CASP7, CCND1, **CD200**, CEACAM1 (includes others), CEBPB, **DGKZ**, EBI3, HLA-F, HMOX1, HRAS, IFNAR2, IL8, IRF2, **LEP**, MAPK13, Mek, **MMD**, NR1H4, NR5A2, NRIP1, PLG, PRDM1, PSMB10, SRC, STAC, STAT5A, TAP1, TAPBP, TNF, TYK2	Inflammatory Response, Inflammatory Disease, Hematological System Development and Function
16	**ADRB1,** ADRB2, AKT1, CAV1, CCNA2, CDKN1B, CDKN2A, **CETN2**, COPS5, DLG1, E2f, EPAS1, **FHL1**, FYN, HIF1A, **HIST1H2AG (includes others)**, **MBNL2**, MDM2, NUP153, PAK3, **PPM1A**, PRL, **PTPN11**, **RAD23B**, RBL2, SLC6A15, **STC2**, TCF4, TERT, TFIIH, TP53, **UCHL1**, VCAN, XPC, ZNF217	Cancer, Genetic Disorder, Respiratory Disease
15	ADCY, **ANGPT1**, **ATP9A**, CD55, CTNND1, CXCR4, CYFIP2, **DMD**, ERK1/2, F7, FSH, GNB1, **GNG2**, GRLF1, hCG, **HSD11B1**, ITGA3, ITGA4, ITGB5, JMJD6, **LEP**, Lh, LIF, Mek, PLAU, PLAUR, **PTP4A1**, RASAL2, RGS12, SH3BP4, **SNCG**, TNFRSF11B, TNFSF11, TRO, VTN	Cell-To-Cell Signaling and Interaction, Tissue Development, Cellular Movement
13	**AKR1C1/AKR1C2**, BAX, **BCL2L13**, BUB1B, **C13orf15**, CASP3, Cbp, CCNA2, CDH2, CDK1,	Cell Death, Renal Necrosis/Cell Death,

	**CRYAB**, **DAAM1**, **GLRX**, HSPB1, **HSPB8**, HTT, Interferon alpha, ITGAV, ITGB3, Jnk, KDM5B, **KIF5B**, **LIMCH1**, MAP3K5, MAPK8, **MT1F**, NF2, P38 MAPK, PKMYT1, RAC1, TGFB1, TNFSF10, TP73, VEGFA, **ZNF124**	Embryonic Development
Extra prostatic cancer		
17	ATF3, BCL3, **DAAM1**, DDIT3, **DZIP3**, E2F1, EGFR, ERBB2, ERK1/2, **FADS1**, HIC1, HIF1A, IL6, IL8, ITGA2, JUN, KDM5B, Lh, MBD3 (includes EG:17192), **mir-30**, **mir-143**, PRDM5, **PSIP1**, RELA, **S100A2**, **SCD**, SMARCA4, SP1, SUZ12, **TCEANC**, TGFB1, TGFB2, **TNS3**, TP53, TP73	Cell Cycle, Cell Death, Cancer
15	ADCY, **AGT**, ALB, ALOX15, **BTC**, CCR5, **CYSLTR2**, E2F1, EGR1, EIF4EBP1, ERK1/2, IFNG, IL4, IL6, IL8, IL24, IL1B, IL1R1, IRAK1, **IRAK2**, ITGB3, **MEIS1**, MYD88, NOS2, **NPY1R**, **NPY5R**, **ORM1/ORM2**, PPARG, **PTN**, PYY, SERPINE1, STAT1, TRAF6, USF1, VEGFC	Cellular Growth and Proliferation, Inflammatory Response, Organismal Injury and Abnormalities

Down-regulated molecules are in bold and color green, whereas up-regulated are red
