# Supplementary figures and images for: Obesity and prostate cancer: gene expression signature of human periprostatic adipose tissue
Source: BMC Med. 2012 Sep 25;10:108. doi: 10.1186/1741-7015-10-108 (PMC3523039; doi:10.1186/1741-7015-10-108)

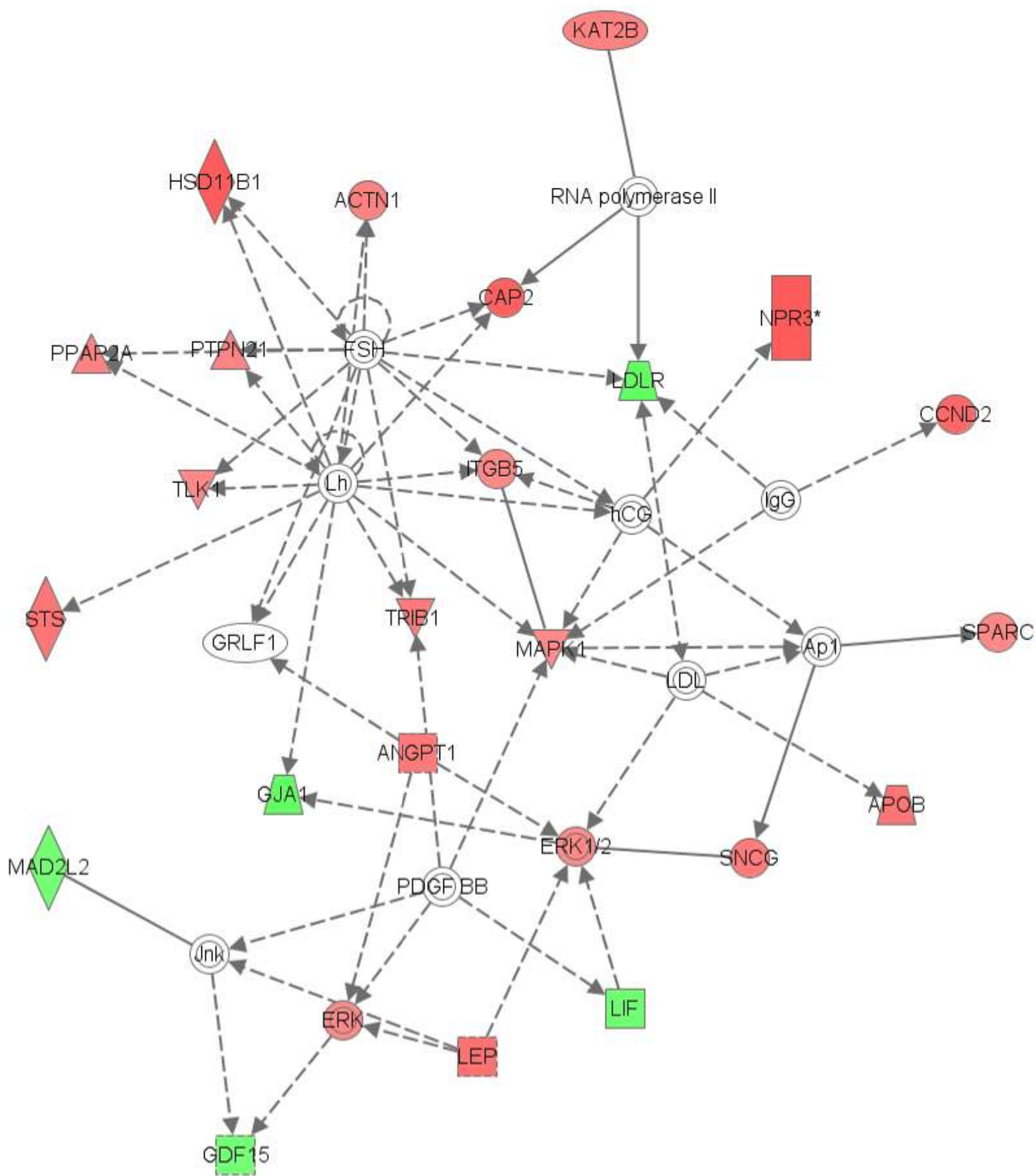

Supplement: Additional file 2 — , Figure S1. Representative network and genes differently expressed in OB/OW versus lean in IPA analysis. Genes are represented as nodes and the biological relationship between two nodes is represented as an edge line. Uncolored genes were not identified as differently expressed in our experiment even though they are relevant to this network. Node shape indicates enzymes (rhombus), phosphatases (triangle), kinases (inverted triangle), G-protein coupled receptor (rectangle), growth factor (square), transporter (trapezoid), transcription factor (ellipse), other (circle). [file 1741-7015-10-108-S2.PDF]
