# Supplementary material for: Obesity and prostate cancer: gene expression signature of human periprostatic adipose tissue
Source: BMC Med. 2012 Sep 25;10:108. doi: 10.1186/1741-7015-10-108 (PMC3523039; doi:10.1186/1741-7015-10-108)
Supplement: Additional file 3 — , Table S2. Significant functions with altered networks and molecules in PP adipose tissue of EPCa versus BPH subjects. [file 1741-7015-10-108-S3.PDF]

Table S2. Significant functions with altered networks and molecules in PP adipose tissue of EPCa vs. BPH subjects (P<0.0001 for inclusion)

| Score | Molecules in Network | Top functions |
|---|---|---|
| *All subjects* | | |
| 32 | ALPL, **BECN1**, CCL8, **CCNC**, CIITA, DDX58, **EBAG9**, ERK1/2, FSH, GPR56, Histone h3, HLA-DRA, HLA-DRB1, **HOXA9**, **IDE**, IL1F9, LGALS8, Lh, MAP2K3, **MAPK8IP1**, MT2A, **NAE1**, NFkB (complex), NLRP4, **PPP2R5C**, **PRKD1**, **RAB4A**, **RBBP8**, RNA polymerase II, **SMAD5**, SMAD7, TLN1, TNFRSF4, **TNFSF4**, **TP53BP2** | Cell Death, Cellular Growth and Proliferation, Hematological System Development and Function |
| 17 | AGTR1, BCL2L1, EFTUD2, ELMOD3, EPO, FANCC, FN1, FOXO3, GAB1, **GULP1**, HNRNPM, INSR, **MYOC**, NR3C1, **OAT**, p85 (pik3r), PDE3B, **PLCB1**, PRKCI, RHOA, SART1, **SCHIP1**, **SMNDC1**, SNRNP200, **SNX7**, SREBF1, **SUMO2**, TF, TFRC, TNFRSF21, **TNFSF4**, YWHAG, **YWHAH**, YWHAQ, **ZBTB1** | Cell Cycle, Endocrine System Disorders, Gastrointestinal Disease |
| 14 | BIRC5, BMI1, CADM1, CASP8, CASP9, CCL19, **CCNG1**, **CCRL1**, CD44, **CD276**, CDC7, **CLCA2**, CLDN7, CLRN1, CREBBP, CXCL10, **DLC1**, E2F3, **EPCAM**, HOXB6, HOXB9, IFN Beta, IFNA4, IRF1, IRF3, IRF7, ITGB4, MCM3, MCM4, MCM7, PLK1, ST18, TCF7L2, TP53, **TSPAN8** | Cell Death, Cellular Growth and Proliferation, Cancer |
| 14 | Actin, ANG, **ANTXR1**, **ARHGEF3**, CASP1, CASP3, CCL5, **CFHR1**, **CREB5**, **ENPP2**, Fibrinogen, GAB1, HMGA1, **IDI1**, IL6, IL13, IL6R, **IL6ST**, JAK1, JAK2, KIT, **let-7**, NPC2, Ras, RNASE1, RNASE2, S100A8, S100A9, **SWAP70**, **THNSL2**, **TMOD3**, TUBB2C, VASP, VCL, WNT5A | Cellular Movement, Immune Cell Trafficking, Cellular Development |
| 12 | Alp, BMP2, CCNA2, **CD24**, **CD34**, COL18A1, DDIT4, E2F3, EGF, F2, FGF2, FN1, FOXO1, GADD45B, GDF6, GLI1, **GRIA3**, **mir-221**, mir-331, MIS12, MYB, NDC80, **NSL1**, PLA2G4A, PRDM5, **S100A10**, SELP, SERPINE1, SMAD4, **SNRNP48**, TGFB1, TGFBR2, TMEM79, WISP2, ZWINT | Cellular Growth and Proliferation, Skeletal and Muscular System Development and Function, Cardiovascular System Development and Function |

| | | |
|---|---|---|
| 11 | ABCA1, BIRC2, CD40, CD40LG, CFLAR, EBI3, ETS1, **FABP3**, FADD, **HLA-DQA1**, **HLA-DQA2**, HLA-DR, IGFBP2, IL27, IRF1, LATS1, **LIN7A**, **MBD4**, PPARD, RASSF1, REL, **RIPK2**, **ROBO1**, **SAV1**, **SNTB1**, **STAC**, STK3, TAB2, **TDP2**, TNF, TNFAIP3, TNFRSF1B, TRAF2, TRAF3, TRAF5 | Cell Death, Cellular Growth and Proliferation, Hematological System Development and Function |
| 11 | **ADAMTS5**, **AHNAK2**, BCAR1, **C14orf45**, **CAMK2N1**, CaMKII, CARD16, **CASP1**, CCL21, CCND1, **DLL4**, EPO, ERK, **FYB**, HIC1, IL18, LAMA3, LGALS1, MAP2K3, Mek, NR4A2, NTRK3, PI3K (complex), POU2F1, PTK2B, PXN, Ras, **RIPK2**, **SHC3**, SKAP1, **SNAPC1**, SRC, **TLE1**, TLR4, TRIP6 | Cellular Movement, Cell Death, Inflammatory Response |
| 11 | **CD9**, CD82, CTNNB1, **DEGS1**, EIF4A1, EZH2, FBL, **GPA33**, GPR56, HDAC1, HDAC2, IgG, KLF4, KLK3, NME1, NOP58, NUFIP1, **ODC1**, PABPC1, **PAIP1**, **PLEKHA5**, RAD9A, RNU4-1, **RPAP3**, RUVBL1, RUVBL2, **SVIL**, TGFBR2, TH, **TOPBP1**, **TSHZ3**, **UGCG**, WNT1, WNT5A, YWHAG | Cellular Movement, Cell Cycle, Cellular Development |
| 6 | CD3, **CD74**, CD247, ERK1/2, **FFAR2**, FHL2, FN1, **GMNN**, HAVCR1, IFN Beta, IFNG, IL4, IL8, ITGA3, **ITGA11**, ITGB1, ITK, JUN, LAT, LCK, LGALS1, LGALS3, Lh, NCOR2, P38 MAPK, PI3K (complex), PTK2B, PTPRC, PXN, **SH2D2A**, SMARCA2, SMARCA4, **SPTB**, TP53, ZAP70 | Cell-To-Cell Signaling and Interaction, Hematological System Development and Function, Immune Cell Trafficking |
| *Lean subjects* | | |
| 22 | ABCA1, **CLEC4E**, **CXCR5**, **DYNLT3**, Estrogen Receptor, FOXP3, **FYB**, GRB2, **HLA-DRA**, IL13, IL1B, ITPR1, **LIN7A**, Mapk, **MEFV**, **mir-21**, **MT2A**, NCK1, NFkB (complex), **NGFR**, **PIK3C2B**, **PLCXD1**, PTK2B, PYCARD, RFX5, **SH2D2A**, SKAP1, SKAP2, Sos, TGFBR2, TIMP3, TNF, TNFRSF11B, TRADD, WAS | Inflammatory Disease, Gastrointestinal Disease, Genetic Disorder |
| 20 | BIRC5, CASP3, CDC25A, CDC25C, **CDK1**, CTNNB1, Cyclin B, DLGAP5, E2F2, E2F3, E2F4, FEN1, GADD45A, **GADD45B**, **GLRX**, ITPR1, KDM5B, **let-7**, LZTS1, MSH2, **MT1A**, **MT1B**, **MT1E**, **MT1F**, MYC, MYCN, SATB1, SFN, **SLC25A19**, SLC9A3R1, **TNFRSF10C**, TNFSF10, TP73, **TSPAN8**, **UBE2T** | Cell Cycle, Cancer, Connective Tissue Development and Function |

| | | |
|---|---|---|
| *OB/OW subjects* | | |
| 17 | **ADM**, APPL1, CASP8, **CCNG1**, **CD9**, **CD151**, **CHRM3**, CLCA2, CTNNB1, EZH2, **GMEB1**, GPR56, **HOXA9**, IgG, Integrin alpha 3 beta 1, **KCNIP3**, KIF23, KLK2, KLK6, **LIN9**, **MST1**, NOP58, **NRCAM**, PSEN1, RNU4-1, **RPAP3**, RUVBL2, **SERPINA3**, SMPD1, TP53, **UGCG**, VCAN, WNT1, WNT5A, ZDHHC2 | Cell Morphology, Tissue Development, Cancer |
| 15 | **ADM**, **AKT3**, ATF1, **BBC3**, CALCA, CASP1, **CASP12**, CD40, **CD44**, CYBB, DIABLO, ELAVL1, **FABP3**, **FCGR2B**, FOXP3, IFNG, IL4, IL22, IL24, **IL13RA1**, MAPK14, MUC5AC/MUC5B, NOD2, PLA2G2A, PPARD, **PPP2R1B**, **RIPK2**, **SCUBE1**, SELE, SELP, SOD2, **STAC**, TNF, TNFRSF1B, TRIP6 | Inflammatory Response, Cellular Movement, Hematological System Development and Function |
| 15 | Actin, Alp, **ANTXR1**, BIRC5, CASP3, CCNA2, **CD34**, COL18A1, Collagen(s), **CORO2B**, CREBBP, EP300, F3, FASLG, FGF2, FN1, **GDF6**, **HOXB6**, ITGB2, **KRT81**, **let-7**, **mir-331**, **MLXIPL**, MYB, MYBL2, **NPAS2**, PCNA, **RHOD**, SELE, SELP, **TFPI**, TGFB1, THBS1, **TMOD3**, VCL | Cell-To-Cell Signaling and Interaction, Tissue Development, Cellular Growth and Proliferation |
| 14 | BRCA1, CD40, **CHIC2**, **EBAG9**, ELAVL1, EPO, FANCC, FN1, GAB1, IL1B, MAP3K14, MAPK1, MCL1, **MYOC**, **NAE1**, NR3C1, **OAT**, **PCLO**, **PLEKHA5**, PTGS2, RELA, RHOA, RNA polymerase II, **RPL4**, **SNX7**, SREBF1, **TDP2**, **TF**, TFRC, TNFRSF1B, **TP53BP2**, TRAF2, TRAF3, TRAF5, YWHAG | Gene Expression, Cell Death, Cell Cycle |
| 14 | AGTR1, **AHNAK2**, ATP2A2, CCL5, CD55, CDKN1C, **CSDA**, E2f, FSH, GPR56, hCG, HIC1, ITGA3, Lh, MAPK1, MAPK8, MMP2, NOTCH1, PIK3CD, Pka, **PLA2G10**, **PLCB1**, **PPP2R5C**, **PSIP1**, **SACS**, **SNAPC1**, THBS1, TLN1, **TRAM2**, **TRO**, TRPC3, **TUBB2C**, VCL, VEGFC, **WDR76** | Cell-To-Cell Signaling and Interaction, Tissue Development, Cancer |
| 14 | 26s Proteasome, Actin, **ANKRD36B (includes others)**, APP, BAX, BRCA1, **C13orf15**, **CD44**, CDK1, CDT1, CHD4, CTGF, **FADS3**, **FFAR2**, **GLRA1**, **GMNN**, HDAC1, HDAC2, Histone h4, **IVNS1ABP**, Jnk, KDM5B, NCOR2, **PDE3B**, **PSMB5**, PTGS2, REST, SIN3A, SMARCA2, | Cell Cycle, DNA Replication, Recombination, and Repair, Gene Expression |

| | | |
|---|---|---|
| | SMARCA4, SMARCB1, **SMC5**, SP1, TFAP4, **TSHZ3** | |
| 12 | **ABCC4**, **BCAP31**, CANX, **CD44**, **CD276**, **CFHR1**, CLDN7, CLRN1, **EPCAM**, ERK1/2, **F10**, F2R, FGF2, Fibrinogen, FN1, Focal adhesion kinase, GRB2, **H19**, HAMP, HFE2, HMMR, ITGAM, ITGB2, **LGALS8**, MAPK14, MET, MMP2, MMP7, MMP14, **NEO1**, OSM, Pka, SPP1, **TSPAN8**, VEGFA | Cell-To-Cell Signaling and Interaction, Tissue Development, Cellular Movement |

Altered molecules are bold. Down-regulated are in green color, whereas up-regulated are red.
